# Frequency Analysis of EEG Microstate Sequences in Wakefulness and NREM Sleep

**DOI:** 10.1007/s10548-023-00971-y

**Published:** 2023-05-30

**Authors:** Milena C. Wiemers, Helmut Laufs, Frederic von Wegner

**Affiliations:** 1 Department of Neurology and Clinical Neurophysiology, Lüneburg Hospital, Bögelstrasse 1, 21339 Lüneburg, Germany; 2 Department of Neurology, Christian-Albrechts University Kiel, Arnold-Heller-Strasse 3, 24105 Kiel, Germany; 3 School of Biomedical Sciences, University of New South Wales, Wallace Wurth Building, Kensington, NSW 2052 Australia

**Keywords:** Electroencephalography (EEG), EEG microstates, NREM sleep, Oscillations, Entropy, Information theory

## Abstract

The majority of EEG microstate analyses concern wakefulness, and the existing sleep studies have focused on changes in spatial microstate properties and on microstate transitions between adjacent time points, the shortest available time scale. We present a more extensive time series analysis of unsmoothed EEG microstate sequences in wakefulness and non-REM sleep stages across many time scales. Very short time scales are assessed with Markov tests, intermediate time scales by the entropy rate and long time scales by a spectral analysis which identifies characteristic microstate frequencies. During the descent from wakefulness to sleep stage N3, we find that the increasing mean microstate duration is a gradual phenomenon explained by a continuous slowing of microstate dynamics as described by the relaxation time of the transition probability matrix. The finite entropy rate, which considers longer microstate histories, shows that microstate sequences become more predictable (less random) with decreasing vigilance level. Accordingly, the Markov property is absent in wakefulness but in sleep stage N3, 10/19 subjects have microstate sequences compatible with a second-order Markov process. A spectral microstate analysis is performed by comparing the time-lagged mutual information coefficients of microstate sequences with the autocorrelation function of the underlying EEG. We find periodic microstate behavior in all vigilance states, linked to alpha frequencies in wakefulness, theta activity in N1, sleep spindle frequencies in N2, and in the delta frequency band in N3. In summary, we show that EEG microstates are a dynamic phenomenon with oscillatory properties that slow down in sleep and are coupled to specific EEG frequencies across several sleep stages.

**Supplementary Information:**

The online version contains supplementary material available at 10.1007/s10548-023-00971-y.

## Introduction

The electroencephalographic (EEG) classification of wakefulness and sleep is based on EEG frequency content, specific EEG waveforms (vertex sharp waves, sleep spindles, K-complexes), and distinguishes the following vigilance states: wakefulness, non-rapid eye movement (NREM) sleep stages N1-N3, and rapid eye movement (REM) sleep (American Academy of Sleep Medicine 2007). In this study we analyze time series properties, and especially oscillatory characteristics of EEG microstate sequences in wakefulness and NREM sleep stages.

EEG microstates are transient, quasi-stable electric field topographies as measured by surface EEG and identified via a clustering algorithm. Microstate sequences are time series representations of EEG data sets which use the reduced set of microstates rather than the full multi-channel data set. In these sequences, the microstate labels appear in contiguous blocks that last tens to hundreds of milliseconds (Koenig et al. 2002; Lehmann et al. 2005; Brodbeck et al. 2012). The clustering principle is to group EEG topographies with high spatial similarity, in order to achieve a maximum percentage of explained spatial variance with respect to the underlying EEG data set. The optimum number of clusters can be determined by a variety of cost functions. In many studies, an optimum of four clusters has been found, and the microstate maps found are highly concordant (Michel and Koenig 2018). This highly reproducible, or canonical set of four maps is usually labelled by the letters A–D (Koenig et al. 2002; Khanna et al. 2015). The polarity of these maps follows the general pattern: left occipital to right frontal (map A), right occipital to left frontal (map B), occipital to frontal (map C), and a more variable map D with a polarity between map C and a radially symmetric pattern. In different studies, the global explained variance (GEV) of these four maps ranged between 58% and 84% (Michel and Koenig 2018).

In the past decades, microstate properties have been characterized for many experimental conditions, most of them for the wakeful state. Microstate properties have been studied in health and in diverse neuropsychiatric diseases such as schizophrenia (Koenig et al. 1999; Lehmann et al. 2005; Perrottelli et al. 2021), early psychosis (de Bock et al. 2020; Murphy et al. 2020), autism (D’Croz−Baron et al. 2019; Nagabhushan Kalburgi et al. 2020), narcolepsy (Kuhn et al. 2015; Drissi et al. 2016), and different forms of dementia (Dierks et al. 1997; Strik et al. 1997; Schumacher et al. 2019; Smailovic et al. 2019; Tait et al. 2020). Several studies investigated the relationship between microstates and resting-state networks (RSNs) identified by functional magnetic resonance imaging (fMRI) (Britz et al. 2010; Musso et al. 2010; Yuan et al. 2012) or by source localization approaches such as topographic electrophysiological state source-imaging (TESS) (Custo et al. 2017), as both are assumed to reflect spontaneously activating functional brain networks. These studies of the wakeful state suggested an association of microstate classes A and B with cognitive and sensory processing (Milz et al. 2016), and of microstates C and D with the saliency and attention networks, respectively (Britz et al. 2010). The details of these relationships are still debated and are the subject of current research (Seitzman et al. 2017; Michel and Koenig 2018).

Although our knowledge about microstates in sleep is more limited, the four canonical microstates A–D have been found during drowsiness (Comsa et al. 2019; Krylova et al. 2021) and sleep (Cantero et al. 1999; Bréchet et al. 2020), as well. Brodbeck et al. (2012) carried out a microstate analysis of full bandwidth EEG (1–40 Hz) during wake and NREM sleep stages and found highly similar topographies of microstates A–C. Sleep stages N2 and N3 showed an altered map D topography with a more circular (radially symmetric) pattern. Microstate durations increased with deepening sleep stages, accompanied by an increasing time interval between global field power peaks. Both findings suggested a general slowing of microstate dynamics during sleep. Temporal properties were deduced from the microstate transition matrix which captures transitions from one time point to the next, but does not allow statements about higher-order correlations, including oscillations. We have described oscillatory microstate dynamics using a time-lagged mutual information analysis and have found that microstates occurred at twice the dominant EEG frequency in wakefulness when non-smoothed, raw microstate sequences were considered (von Wegner et al. 2017). For example, EEGs with a dominant 10 Hz alpha activity produced microstate sequences with large mutual information values between microstates separated by intervals of 50 ms (20 Hz), 100 ms (10 Hz), and multiples thereof. Sleep EEG shows characteristic oscillatory phenomena such as theta rhythms during drowsiness, sleep spindles in N2, and slow wave delta rhythms in deep sleep (N3) (American Academy of Sleep Medicine 2007), but we do not know whether the oscillatory microstate dynamics in the wake state also occur in sleep.

Furthermore, information-theoretical analysis of microstate sequences during a design task has shown that the entropy rate (von Wegner et al. 2018) and oscillatory microstate dynamics are modulated by cognitive tasks, and that these metrics indicate the balance between cognitive workload and cognitive control (Jia et al. 2021). Yet, we do not know whether this interpretation is compatible with our understanding of reduced cognition during sleep.

Based on our previous results, the aim of this study was to test microstate sequences for their information content and periodic patterns in different sleep stages. Interpreting microstates as electrophysiological correlates of large-scale network activity, we hypothesized that (i) wakefulness-related microstate frequencies would disappear as wake-specific network activity recedes during the wake-sleep transition, (ii) with the appearance of sleep-specific oscillatory phenomena, microstate oscillations in sleep-related frequency bands might appear.

Other recent studies have established relations between microstates and the EEG frequency spectrum (Shi et al. 2020; Abreu et al. 2021) by averaging the instantaneous EEG frequency over epochs assigned to a given microstate. It should be emphasized that there is a key difference between those approaches and this study. The previous approaches identify which frequencies can be found at the EEG sensor level during a given microstate, but they do not describe the temporal ordering of the microstates themselves. Our approach identifies whether the sequence of microstates has periodic properties and is therefore able to track the temporal behavior of large-scale network activity. We use the time-lagged mutual information function (autoinformation function, AIF) of the microstate sequence as a generalized version of the autocorrelation function (ACF), and compare it to the ACF of EEG sensor signals. Via the one-to-one correspondence between ACFs and power spectral densities (Wiener-Khintchine theorem, Wiener 1930; Khintchine 1934; Kubo et al. 1985), EEG spectral peaks can thus be compared to microstate AIFs.

## Materials and methods

### Data

We analyzed EEG recordings of 32 healthy subjects (age range: 19–27 years, mean: 23 years, 20 females, 12 males) participating in a task-free simultaneous EEG-fMRI study (Brodbeck et al. 2012; Jahnke et al. 2012; Gärtner et al. 2015). We included subjects that reached at least sleep stage N2 and with a minimum duration of 105 s in each vigilance state. A total of 19/32 subjects reached sleep stage N3. Wakefulness represents a task-free condition with eyes closed.

For each subject, data segments from different vigilance states were adjusted to have identical length. Across subjects, data sets had a total duration between 105 and 210 s (mean: 157.97 s). The EEG pre-processing steps have been detailed in our earlier studies (Brodbeck et al. 2012; Jahnke et al. 2012) and included: correction of MR gradient and ballistocardiogram artifacts, down-sampling to 250 Hz and re-referencing to an average reference. In this study, signals were band-pass filtered to 0.5–20 Hz, using a 6th order digital Butterworth filter.

### Sleep Scoring

Sleep stages were assigned according to the criteria defined by the American Academy of Sleep Medicine (American Academy of Sleep Medicine 2007). Sleep graphoelements (SGE) including sleep spindles were marked manually. Microstate sub-sequences from each subject corresponding to sleep spindles in sleep stage N2 were also analyzed independently from the surrounding N2 data. Sleep spindle segments (n = 32) had durations ranging from 544 ms to 1844 ms (mean: 951.4 ms).

### Spectral Analysis

To summarize the EEG frequency content across all channels, each data set was submitted to a principal component analysis. Only the first component was retained and its power spectral density (PSD) was computed using Welch’s method (Hann window, 4096 ms) (Welch 1967). The relative power of each frequency band was determined as the area under the PSD covered by that frequency band, divided by the total PSD area.

The PSD is the frequency domain equivalent of the autocorrelation function (ACF) in the time domain. The ACF measures the linear correlation between the signal ${x}_{t}$ at time point *t* and the same signal at time point *t+*$\tau$. The ACF coefficient $r\left(\tau \right)$ for time lag $\tau$ is defined as the expected value $E\left({x}_{t}{x}_{t+\tau }\right)$. Oscillatory signals with frequency *f* have a periodic autocorrelation with oscillation length *Τ = 1/f*. For example, a pure 10 Hz oscillation (idealized EEG alpha-rhythm) has local ACF maxima at multiples of 100 ms and local ACF minima at odd integer multiples of 50 ms (50, 150, … ms).

### Microstate Analysis

**Fig. 1:**
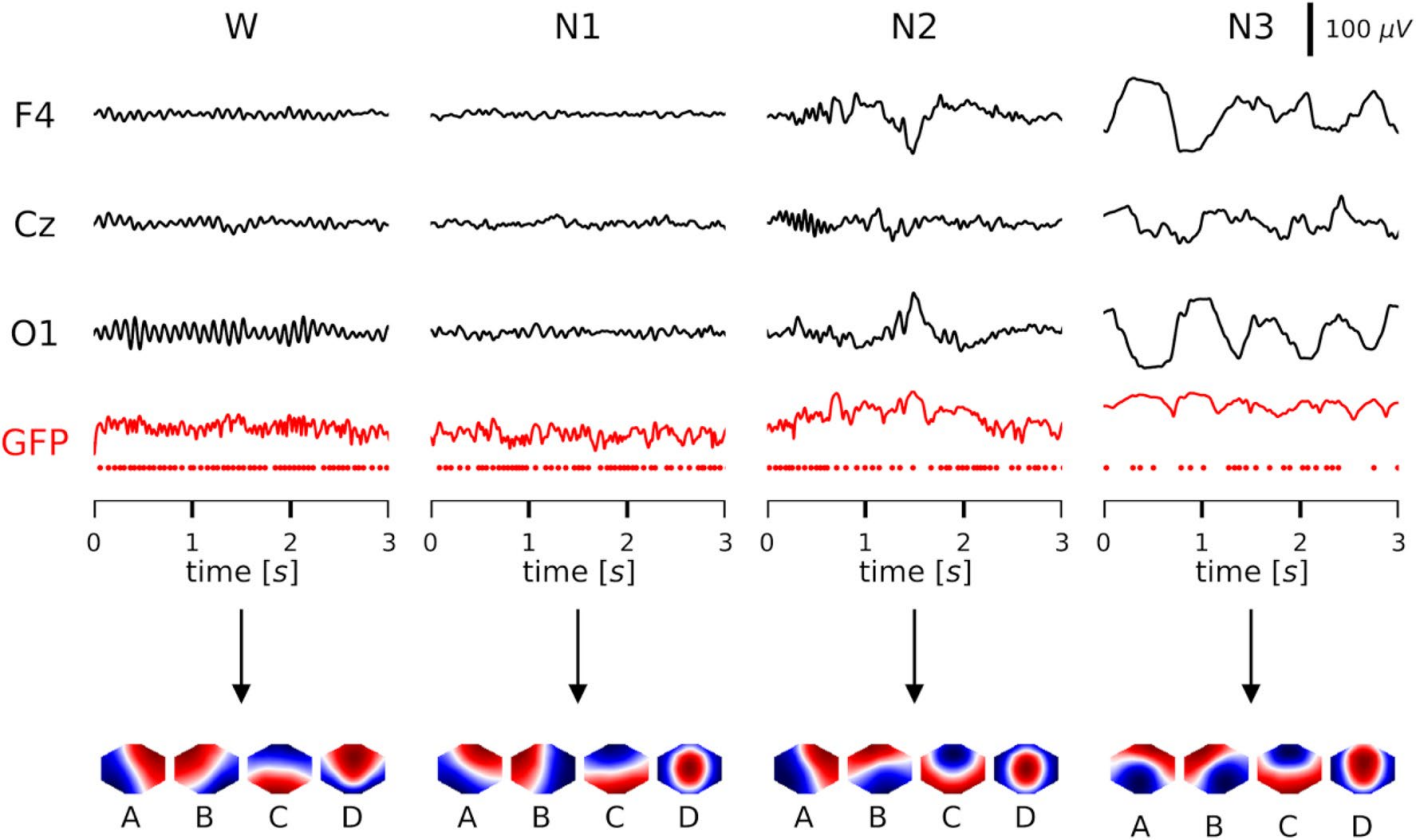
EEG microstates in wakefulness (W) and NREM sleep stages N1–N3. The four vigilance states are illustrated by exemplary EEG traces (3 s) from three exemplary channels (F4, Cz, O1; black). Wakefulness is characterized by occipital alpha oscillations (8–12 Hz). Sleep stage N1 has low-amplitude mixed frequency (LAMF) activity, N2 shows a sleep spindle (first second at Cz) followed by a K-complex and N3 is characterized by generalized delta waves (0.5–3 Hz). Global field power (red line) and GFP peaks (red dots) are shown below the EEG traces. The microstate group maps A–D of each vigilance state are shown below the GFP time series (note that their polarity can be ignored)

For each multichannel EEG data set, the spatial standard deviation, or global field power (GFP), is computed at each time point (Lehmann and Skrandies 1980):

1 $$GFP\left(t\right)=\sqrt{\frac{1}{N}{\sum }_{i=1}^{N}{v}_{i}^{2}\left(t\right)}$$

where ${v}_{i}\left(t\right)$ are the average-reference potentials measured at $N$ electrodes. Figure 1 shows representative EEG waveforms from three exemplary EEG channels for each vigilance state, and the associated GFP time series (red). At each local maximum of the GFP function, the global electric field topography is assumed to be stable (Lehmann et al. 1987; Zanesco 2020). The voltage distributions (topographies) at these local GFP maxima are the input data for the modified k-means algorithm (Pasqual–Marqui et al. 1995), that we implemented in the Python programming language (von Wegner and Laufs 2018). The computed cluster centroids are the *K* microstate maps for the given subject. As in our previous analysis of the sleep EEG data set, we used a clustering into *K* = 4 microstate classes (Brodbeck et al. 2012). The k-means algorithm was run five times and the result explaining most of the spatial variance was selected for each subject and sleep stage. From the individual subject maps, we calculated group maps via the full permutation procedure described in Koenig et al. (1999), using 20 independent runs and retaining the result with the maximum explained variance. Microstate maps were computed for each vigilance state separately. Results for microstate sequences based on K = 5 microstate classes and two different clustering levels (per sleep stage, grand mean maps across all sleep stages) are found in the supplementary material.

Microstate sequences were obtained through back-fitting of the group maps into the original EEG data sets. At each time point, the microstate class label *i ∈ {A, B, C, D}* that best fitted the current EEG topography was used, as measured by the squared correlation coefficient between the microstate map and the EEG topography (Koenig et al. 1999; Murray et al. 2008). To keep the temporal structure of the microstate sequence unaltered, back-fitting was performed at each time point, as opposed to the frequently used technique of fitting at GFP peaks only (Lehmann et al. 1987). No further temporal smoothing methods were applied.

### Microstate Statistics

We evaluated the following parameters for each subject and each vigilance state:

- GFP peaks per second (PPS): Mean number of local GFP maxima per second [1/s].
- Mean microstate duration (MMD): Average time a microstate map remains stable before switching to the next map [ms].
- Global explained variance (GEV): GFP-weighted percentage of spatial variance explained by the maps [%].
- Transition probability matrix: a 4x4 stochastic matrix T which contains the conditional probabilities *T*_*ij*_* = P (X*_*t+1*_ *= j | X*_*t*_ *= i)* to switch from microstate label i to label j in a single time step, with *i, j ∈ {A, B, C, D}*.
- The relaxation time (T_relax_) of the transition probability matrix *T* describes how quickly the process described by *T* relaxes from perturbations. It is the inverse of the spectral gap, which is defined as the difference between the two largest eigenvalues of *T*.

Statistical differences were assessed with one-way ANOVA tests across vigilance states followed by post-hoc Tukey tests (*α = 0.05*).

### Markov Surrogate Data

The distribution of microstate labels will be denoted by $\pi$ and describes the probability that the microstate sequence *X*_*t*_ has label *i ϵ {A, B, C, D}* at time point *t*, in mathematical notation $P({X}_{t}= i)$. The microstate distribution $\pi$ and the transition probability matrix (${T}_{ij}=P({X}_{t+1}= j|{X}_{t}= i)$) allow the construction of surrogate sequences with a purely Markovian structure (Häggström 2002; von Wegner et al. 2017). To detect deviations of EEG microstate sequences from Markov processes, we tested empirical microstate sequence properties against null hypothesis distributions from surrogate data. For each vigilance state, we averaged *T* and *π* from the empirical microstate sequences of all subjects. Confidence intervals were computed from *n* = 500 Markov surrogates at significance level *α = 0.05*.

### Entropy-Related Quantities

To characterize the amount of information (or randomness) contained in microstate sequences, and to describe their autocorrelation structure in an information-theoretic sense and independent of the label assignment, we analyzed two entropy-related quantities, (i) the finite entropy rate, and (ii) the autoinformation function (AIF).

Both quantities are based on the microstate label distribution. The microstate sequence can be characterized by its Shannon entropy (Kullback 1959):

2 $$H\left(X\right)=-{\sum }_{i\in \left\{A,B,C,D\right\}} P\left({X}_{t}=i\right){log}_{2}P\left({X}_{t}=i\right)$$

which is minimal (*H(X) = 0*) if the sequence consisted of a single, never changing microstate, and maximal (*H(X) = log(4)*) if each microstate was assigned to 1/4 of all samples. The Shannon entropy is a static measure in the sense that it does not contain any information about the temporal order of the samples.

Time dependence is introduced by using conditional entropies. We use a finite version of the entropy rate (Levin et al. 2006), defined as a conditional entropy:

3 $${h}_{1}=H\left({X}_{t+1}|{X}_{t}^{\left(k\right)}\right)$$

This quantity measures the uncertainty (or entropy) about the next microstate label ${X}_{t+1}$, given knowledge about the *k* previous microstates ${X}_{t}^{\left(k\right)}$. The computation involves the Shannon entropy of multivariate distributions (joint entropy), as detailed in Kullback (1959), Cover and Thomas (2006) or von Wegner (2018). We chose a history length of *k* = 6 samples (24 ms) because entropy rate estimates become less reliable for *k* > 6 for the sequence lengths in this study (von Wegner et al. 2018).

A sequence ${X}_{t}$ with a large entropy rate has a short decorrelation time, or a quickly fading memory of its past values. In other words, if microstate labels are produced with a high level of randomness and low predictability, the entropy rate will be high. A lower entropy rate, on the other hand, means that the next microstate is more predictable from past values. Entropy rate differences were assessed with one-way ANOVA tests across vigilance states followed by post-hoc Tukey tests (*α = 0.05*).

Two-point correlations between the time points *t* and *t+*$\tau$, for an arbitrary time lag $\tau$, are measured by the shared (mutual) information between the microstate labels ${X}_{t}$ and ${X}_{t+\tau }$. Mutual information is a generalization of the linear correlation coefficient between two random variables, and thus, the collection of time-lagged mutual information coefficients is a generalization of the time autocorrelation function (ACF), and will be denoted autoinformation function (AIF) (Cover and Thomas 2006; von Wegner et al. 2017):

4 $$AIF\left(\tau \right)=H\left({X}_{t+\tau }\right)-H\left({{X}_{t+\tau }|X}_{t}\right)$$

The magnitude of the AIF coefficient at time lag $\tau$ indicates how well the microstate at time *t* predicts the microstate at time *t+*$\tau$. In information theoretic language, it expresses how much the uncertainty (randomness) about ${X}_{t+\tau }$ is reduced by conditioning on ${X}_{t}$. In this way, AIF peaks at regular intervals indicate periodic occurrences of microstates, in the same way that ACF local extrema indicate periodic EEG signals.

#### AIF peak test

To test AIF curves for significant peaks (local maxima), we constructed an additional surrogate test. An AIF curve is said to have a significant peak at time lag $\tau$ if the normalized area under the AIF curve (AUC) in a narrow window around time lag $\tau$, i.e. $\left[\tau -w,\tau +w\right]$, is significantly larger than the corresponding area under the surrogate data AIF. Areas were normalized with respect to the total area under the AIF curve.

The statistic testing whether the AIF $a\left(t\right)$ has significant peaks at multiple time lags ${\tau }_{k}$ (*k* = 1, …) is $\frac{1}{A}{\sum }_{k} \int \limits_{\tau_k-w}^{\tau_k+w}a\left(t\right)dt$, with normalization constant $A=\int a\left(t\right)dt$. The value of this statistic was compared to the null distribution obtained from *n* = 10 Markov surrogate values, using the Mann-Whitney U-Test. We used an empirical window half-width of *w* = 8 ms (2 samples).

We expected AIF peaks to coincide with local extrema of the ACF. For the vigilance states W, N1 and N2, we therefore tested the peak locations ${\tau }_{k}$ given by the first two local extrema of the ACF. For N3, only the first ACF minimum fell within the analyzed range (${\tau }_{max}$ = 1000 ms) and was used as ${\tau }_{k}$ .

For instance, an EEG data set with a 10 Hz spectral peak yields an ACF whose first two local extrema are found at 50 ms and 100 ms, hence the AIF peak test used ${\tau }_{1}$ = 50 ms and ${\tau }_{2}$ = 100 ms.

### Markovianity Tests

The transition matrix exclusively captures those features of microstate sequences that can be described by a first-order Markov process. Markov properties describe to what extent time series remember their past. A zero-order Markov process fulfills the null hypothesis that information about the current state ${X}_{t}$ does not affect the transition probabilities to the next state ${X}_{t+1}$. In a first-order Markov process, the transition probability $P\left({X}_{t+1}\right|{X}_{t}$*)* depends on ${X}_{t}$, but not on ${X}_{t-1}$ or earlier samples. Rules for higher order Markov processes follow the same pattern (Kullback et al. 1962). We tested for the Markov properties of order 0, 1 and 2 in each vigilance state as described previously (von Wegner et al. 2017), at a significance level of *α = 0.05* and with Bonferroni correction across the number of subjects.

All methods were implemented in the Python programming language (von Wegner and Laufs 2018).

## Results

### Microstate Statistics

The group maps found in different vigilance states are shown in Fig. 1. The similarities between sleep maps and wakefulness maps, as measured by their Pearson correlation coefficient, are summarized in Table 1. The average similarity between sleep and wake maps was at least 80%, for all sleep stages. The lowest similarities compared with the wake state were found for map D in N1 (62.2%) and N2 (53.8%), and for map A in N3 (71.0%). The dissimilarity of map D can be described visually as being more circular in sleep compared to wakefulness.

**Table 1:** Similarity of group maps in sleep stages N1-N3 as compared to wakefulness [%]

	A	B	C	D	mean
N1	87.9	86.1	93.5	62.2	82.4
N2	99.3	85.4	82.3	53.8	80.2
N3	71.0	92.4	88.6	81.2	83.3

All similarity values were measured by Pearson‘s correlation coefficient.

Table 2 summarizes further microstate properties, presented as mean values and 90% confidence intervals across all subjects. One-way ANOVA tests showed significant differences across vigilance states for all parameters. From left to right, with decreasing vigilance, the number of GFP peaks per second (PPS) decreased, while the mean microstate duration increased. An increasing time interval between subsequent GFP peaks suggests a shift towards lower EEG frequencies, an effect that is explored further in Sect. 3.2.

**Table 2:** Basic microstate properties: GFP peaks per second (PPS), mean microstate duration (MMD) and mean microstate duration per map (MMD _A,B,C,D_), global explained variance per map (GEV_A,B,C,D_) and total global explained variance (GEV_T_) in wakefulness (W) and sleep stages N1-N3.

	W		N1		N2		N3	
	Mean	CI	Mean	CI	Mean	CI	Mean	CI
PPS [1/s]	18.5	(17.0;20.2)	16.6	(15.5;18.0)	15.4	(14.2;16.7)	11.5	(9.3;13.3)
MMD [ms]	21.9	(15.4;29.0)	28.3	(20.7;34.6)	31.4	(24.2;38.9)	53.3	(38.9;74.6)
MMD_A_ [ms]	20.2	(13.2;30.1)	27.7	(22.7;31.8)	31.8	(23.7;39.1)	55.8	(42.0;73.1)
MMD_B_ [ms]	20.6	(15.3;25.3)	27.1	(19.6;33.5)	33.7	(29.7;40.1)	52.3	(37.7;74.3)
MMD_C_ [ms]	23.4	(18.7;28.4)	30.3	(23.9;35.7)	30.4	(25.4;38.0)	51.5	(40.2;68.6)
MMD_D_ [ms]	23.3	(18.2;29.2)	28.2	(22.1;34.0)	29.5	(23.6;36.0)	53.4	(41.7;67.3)
GEV_A_ [%]	13.0	(4.8;28.6)	15.2	(11.3;19.0)	10.5	(3.1;19.5)	15.0	(10.7;20.1)
GEV_B_ [%]	13.5	(6.1;20.9)	13.4	(5.1;28.7)	16.8	(11.6;24.3)	11.7	(6.5;17.7)
GEV_C_ [%]	18.2	(7.5;27.5)	20.9	(14.9;31.9)	26.7	(15.3;39.3)	26.5	(17.6;39.1)
GEV_D_ [%]	14.8	(5.8;22.6)	9.5	(4.4;15.8)	10.8	(5.1;17.2)	14.2	(7.7;18.9)
GEV_T_ [%]	59.5	(51.6;67.2)	58.9	(53.4;64.2)	64.8	(58.2;71.6)	67.4	(60.5;72.9)

mean: arithmetic means across all subjects, CI: confidence intervals (5–95%) in parentheses.

Post-hoc pairwise Tukey tests revealed that PPS values between all vigilance states were significantly different. For MMD values, the only non-significant differences were the comparisons between sleep stages N1 and N2 for microstates A, C and D. GEV comparisons showed that map C explained the maximum percentage of variance in all vigilance states. The total GEV values ranged from 58.9% (N1) to 67.4% (N3). Detailed statistical results are given in supplementary Table S1.

### Relaxation Time and Entropy rate

The relaxation time of the microstate transition matrix showed significant differences between vigilance states in a one-way ANOVA. Mean relaxation times increased with increasing sleep depth (W: 4.5; N1: 5.7; N2: 6.4; N3: 10.8). The opposite trend was observed for the entropy rate, which decreased from wake to deep sleep (W: 0.86; N1: 0.74; N2: 0.69; N3: 0.48 bits/sampling interval). For both parameters, all pair-wise comparisons between vigilance states were significant (post-hoc Tukey tests).

**Fig. 2:**
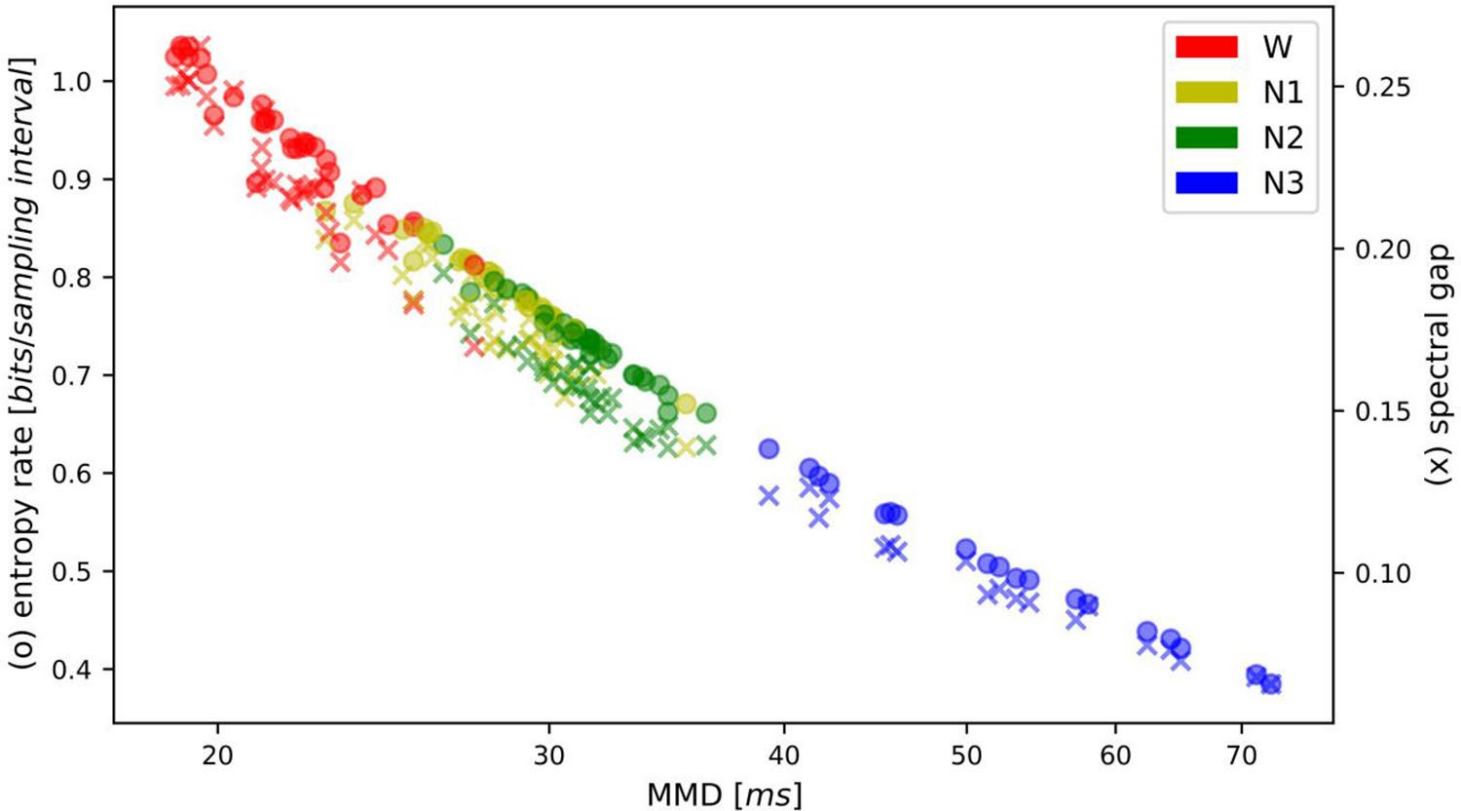
Slowing of microstate sequences with decreasing vigilance. From wake (W) to N3, the mean microstate duration (abscissa) increases, while the entropy rate (left ordinate, circles) and the spectral gap of the transition matrix (inverse of the relaxation time, right ordinate, crosses) decrease. The abscissa (MMD) uses a semi-logarithmic scaling

The relationship between MMD, transition matrix relaxation time and entropy rate is illustrated in Fig. 2. For better visibility, the spectral gap, which is the inverse of the relaxation time, is plotted (right ordinate, crosses). Using a semi-logarithmic scaling for MMD values, the variables entropy rate (left ordinate, circles) and spectral gap decreased almost linearly with increasing MMD, suggesting a near-logarithmic relationship. Spectral gap and entropy rate values decreased at the same rate. Vigilance states are indicated by color and form clearly separated clusters.

### Markovianity

Microstate sequences from wakeful rest and sleep stages N1–N3 were tested for the Markov properties of order 0, 1 and 2. The null hypotheses for Markov processes of order 0 and 1 were rejected for all subjects in all four vigilance states. The null hypothesis for second-order Markovianity was rejected for all subjects in wakeful rest and sleep stages N1 and N2. In sleep stage N3, it was rejected in 9/19 cases. Thus, 10/19 (52.6%) microstate sequences in state N3 could be described as second-order Markov chains. For isolated sleep spindles, zero-order Markovianity was rejected in all 32/32 cases, first-order Markovianity was rejected in 1/32 subject, and second-order Markovianity could not be rejected in any case (0/32).

### Microstate Frequency Analysis

In this section, we test whether microstate sequences contain specific frequencies related to the EEG frequency spectrum. For each vigilance state, we compared the EEG power spectral density (PSD), the EEG autocorrelation function (ACF), and the corresponding microstate autoinformation function (AIF). PSD and ACF curves were computed for the first principal component of each multi-channel EEG data set to summarize all channels into a one-dimensional signal. ACF curves were overlaid onto microstate AIFs for direct comparison of local minima and maxima.

To compare the amount of information stored in a microstate sequence with that of an equivalent first-order Markov process, we added 95% confidence interval AIFs computed from Markov surrogate data.

#### Wakeful Rest

**Fig. 3:**
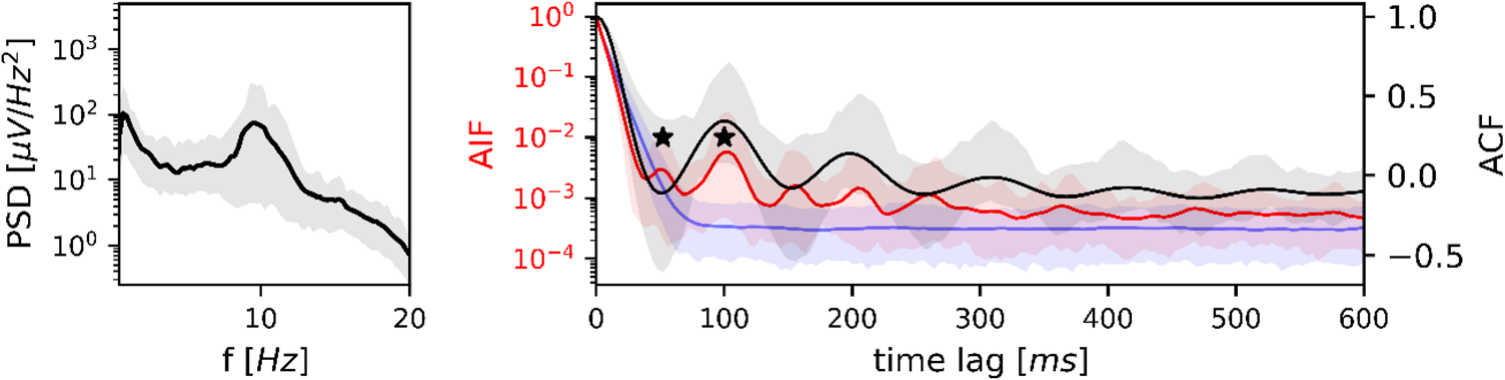
PSD, ACF and AIF of microstate sequences in wakefulness. Left: average power spectral density (*n* = 32) in semi-logarithmic coordinates. Right: average EEG autocorrelation function (black) and microstate autoinformation function (red). Markov surrogate data are shown as the 95% confidence interval (blue-shaded area) and mean surrogate AIF (blue line). The PSD is characterized by an alpha frequency peak at 9.5 Hz, which corresponds to the first local minimum of the ACF at 52 ms (9.6 Hz). AIF peaks at multiples of 52 ms coincide with negative and positive ACF local extrema. The AIF peak test was significant for the first two AIF peaks (marked by asterisks); the first five AIF peaks are significant with respect to the Markov confidence interval while the surrogate AIF does not show any peaks. PSD, ACF and AIF are shown along with their confidence intervals (5–95%)

Wakefulness is analyzed in Fig. 3. The PSD (left) had a frequency peak at 9.5 Hz, within the normal adult alpha frequency range. The relative alpha band (8–13 Hz) power was 41% of the total (0.5–20 Hz) power. The first local maximum of the corresponding ACF (right, black curve) was located at 100 ms, the first local minimum at 52 ms. These two ACF local extrema coincided with the first two local maxima of the microstate AIF (red curve). The first five peaks of the AIF were located outside the confidence interval defined by the Markov surrogate data (blue area), demonstrating that microstate sequences contain a significantly larger amount of shared information at these time lags than the equivalent Markov model. We also applied the AIF peak test described in the Methods section. This test considers the first two AIF peaks and compares them to the mean surrogate AIF (blue curve). It also showed that periodicities were significant (*p < 0.001*) when compared to a first-order Markov model.

#### Sleep Stage N1

**Fig. 4:**
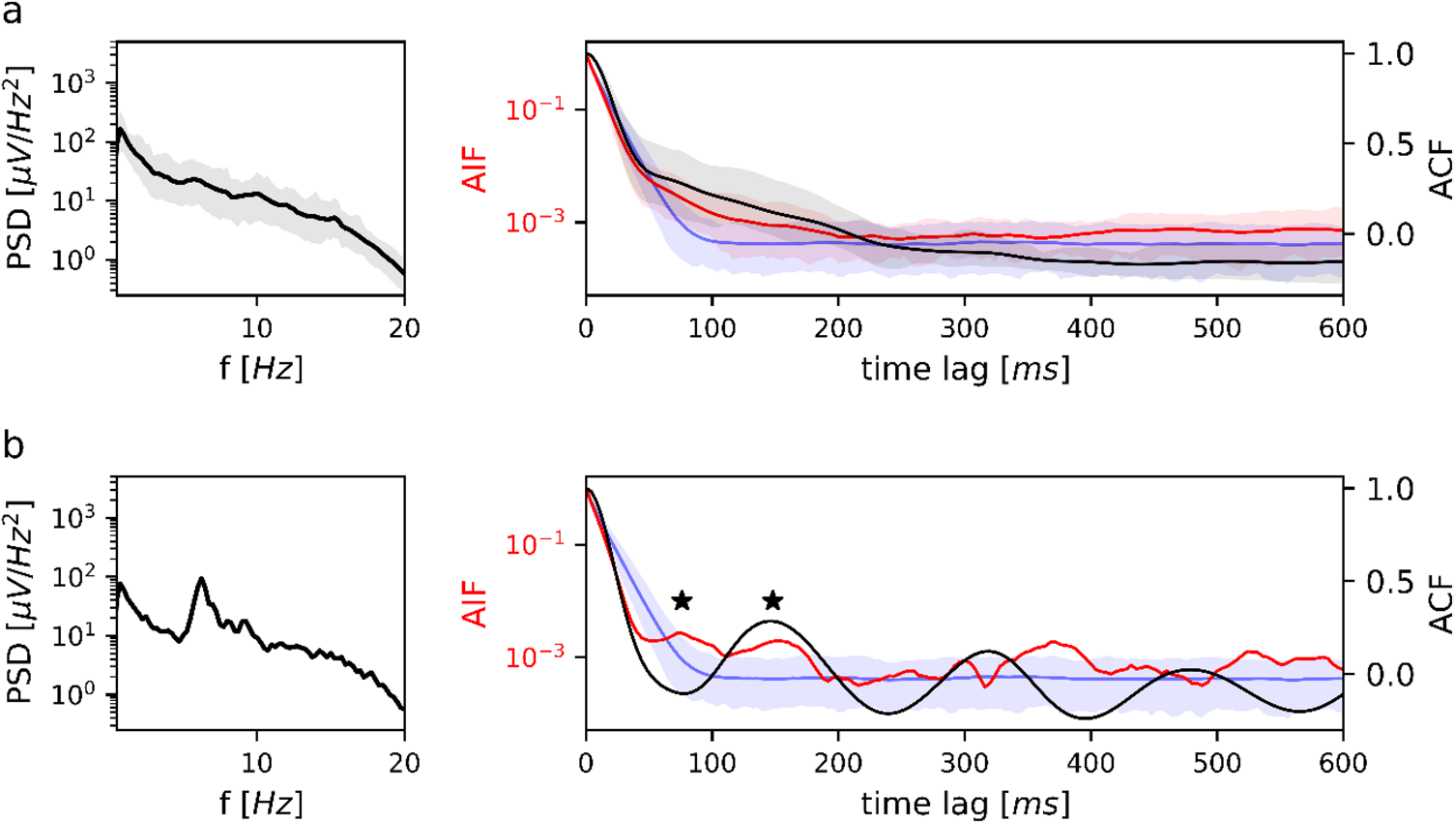
PSD, ACF and AIF of microstate sequences in sleep stage N1. **a** Left: average power spectral density (*n* = 32) in semi-logarithmic coordinates. Right: average EEG autocorrelation function (black) and microstate autoinformation function (red). Markov surrogate data are shown as the 95% confidence interval (blue shaded area) and mean surrogate AIF (blue line). PSD, ACF and AIF do not show clear peaks, indicating the absence of a dominant frequency. PSD, ACF and AIF are shown along with their confidence intervals (5–95%). **b** Results for a single subject with a prominent theta rhythm during N1. The PSD has a 6.3 Hz peak, corresponding to a first local ACF minimum at 76 ms (6.6 Hz). AIF peaks at multiples of 76 ms coincide with negative and positive ACF local extrema. The AIF peak test was significant for the first two AIF peaks (marked by asterisks); they are as well significant with respect to the 95% Markov confidence interval (blue shaded area). AIF peaks do not appear in Markov surrogate data (blue line)

Sleep stage N1 is analyzed in Fig. 4. The group-averaged PSD (Fig. 4a, left) showed a nearly monotone decay without clearly discernible frequency peaks. Accordingly, neither the group averaged ACF nor the AIF showed clear periodicities (Fig. 4a, right). Among all n = 32 subjects, we identified a single subject with prominent theta oscillations during N1. This subject is analyzed in Fig. 4b and showed a PSD spectral peak at 6.3 Hz. The ACF (black curve) had its first two local extrema at 76 ms and 148 ms. For the same time lags, the microstate AIF (red curve) had local maxima outside the confidence interval defined by Markov surrogates (blue area). The AIF peak test described in the Methods section was significant (*p = 0.043*) with respect to the Markov surrogate AIF (blue curve).

#### Sleep Stage N2

**Fig. 5:**
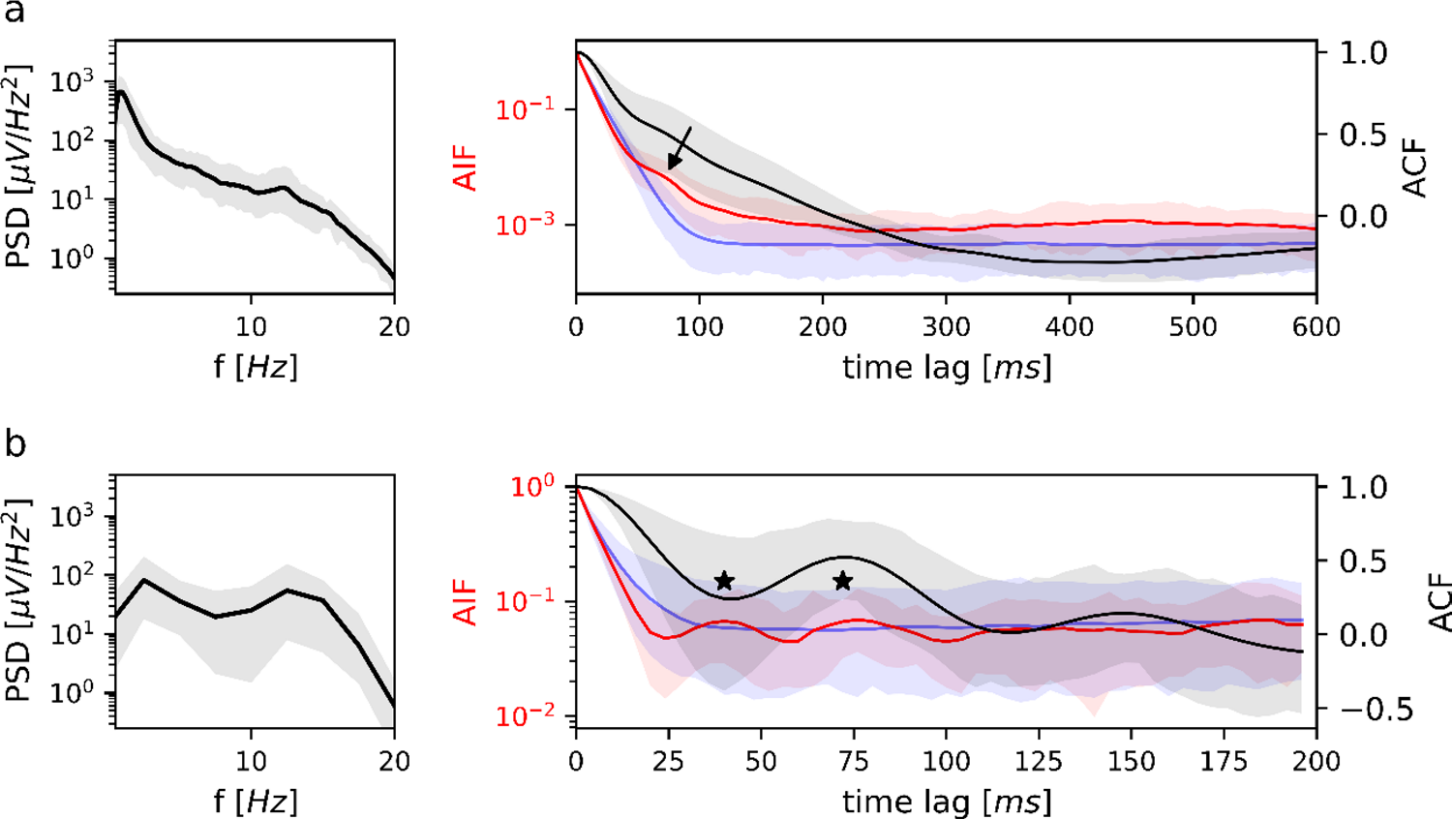
PSD, ACF and AIF of microstate sequences in sleep stage N2. **a** Left: average power spectral density (*n* = 32) in semi-logarithmic coordinates. Right: average EEG autocorrelation function (black) and microstate autoinformation function (red). Markov surrogate data are shown as the 95% confidence interval (blue shaded area) and mean surrogate AIF (blue line). The PSD shows a small peak at 12.2 Hz whereas ACF and AIF show no clear oscillations. The ACF has a ‘shoulder’ (marked by arrow) outside of the Markov confidence interval. The surrogate AIF does not show any peaks. **b** Left: the average PSD for isolated sleep spindles (*n* = 32). Right: average EEG autocorrelation function (black) and microstate autoinformation function (red) for isolated sleep spindles. Markov surrogate data are shown as the 95% confidence interval (blue shaded area) and mean surrogate AIF (blue line). There is a PSD peak at 12.5 Hz, corresponding to the first local ACF minimum at 40 ms (12.5 Hz). AIF peaks at multiples of 40 ms coincide with negative and positive ACF local extrema. Microstate AIF peaks do not exceed the 95% Markov confidence interval but cannot be reproduced by Markov surrogate data (blue line); the AIF peak test was significant for first two AIF peaks (marked by asterisks). PSD, ACF and AIF in (**a**) and (**b**) are shown along with their confidence intervals (5–95%)

Sleep stage N2 results are shown in Fig. 5. The group-averaged PSD for N2 (Fig. 5a) decayed monotonically across the delta, theta and alpha frequency bands. A small peak was observed at 12.2 Hz in the lower beta band, which is a typical sleep spindle frequency. The ACF (black) and AIF (red) curves in the right panel of Fig. 5a did not show clear oscillations. At the time lag corresponding to the 12.2 Hz spindle frequency (82 ms), however, the microstate AIF curve had an elevation above the Markov confidence interval (arrow in Fig. 5a).

To test whether microstates followed spindle frequencies, we analyzed isolated sleep spindle segments. The results are summarized in Fig. 5b; Table 3.

**Table 3:** Sleep spindle analyses: GFP peaks per second (PPS), total global explained variance (GEV_T_) and global explained variance per map (GEV_A,B,C,D_), mean microstate duration (MMD) and mean microstate duration per map (MMD_A,B,C,D_). For comparison, the homologous N2 properties are listed on the right

	Sleep spindles		N2	
	Mean	CI	Mean	CI
PPS [1/s]	19.2	(15.4;22.4)	15.4	(14.2;16.7)
GEV_T_[%]	60.9	(51.6;69.9)	64.8	(58.2;71.6)
GEV_A_[%]	10.4	(2.9;20.6)	10.5	(3.1;19.5)
GEV_B_[%]	16.0	(1.9;29.1)	16.8	(11.6;24.3)
GEV_C_[%]	19.8	(4.4;38.2)	26.7	(15.3;39.3)
GEV_D_[%]	14.7	(1.6;35.6)	10.8	(5.1;17.2)
MMD [ms]	21.1	(12.2;30.8)	31.4	(24.2;38.9)
MMD_A_ [ms]	21.5	(13.4;30.4)	31.8	(23.7;39.1)
MMD_B_ [ms]	20.8	(12.4;27.4)	33.7	(29.7;40.1)
MMD_C_ [ms]	19.4	(11.5;28.8)	30.4	(25.4;38.0)
MMD_D_ [ms]	22.7	(14.0;34.8)	29.5	(23.6;36.0)

mean: arithmetic means across all subjects, CI: confidence intervals (5-95%) in parentheses.

The N2 microstate maps explained 60.9% of sleep spindle segment variance (GEV_T_). One-way ANOVA across microstate classes showed significant differences between the GEV values (*p = 0.004*), while post-hoc Tukey tests revealed that only map A and C differed significantly. We found an average of 19.2 GFP peaks per second (PPS) and a mean microstate duration of 21.1 ms.

Isolated sleep spindles had a spectral peak at 12.5 Hz (Fig. 5b, left), which was reflected by ACF local extrema at 40 ms and 72 ms. AIF peaks appeared at the same time lags, but their absolute magnitude did not exceed the information content of Markovian surrogate data (blue area). Although the AIF corresponding to sleep spindle microstate sequences (red) lies within the Markov surrogate confidence interval (blue-shaded area), its shape is clearly different from the mean surrogate AIF (blue curve). We tested the statistical significance of the apparent peaks in the red curve using the AIF peak test and found that the peaks at time lags 40 ms and 72 ms were significant *(p < 0.001)*. This shows that the AIF peaks of sleep spindle microstate sequences are unique to empirical EEG data. We never observe similar peaks in Markovian surrogate data.

#### Sleep Stage N3

**Fig. 6:**
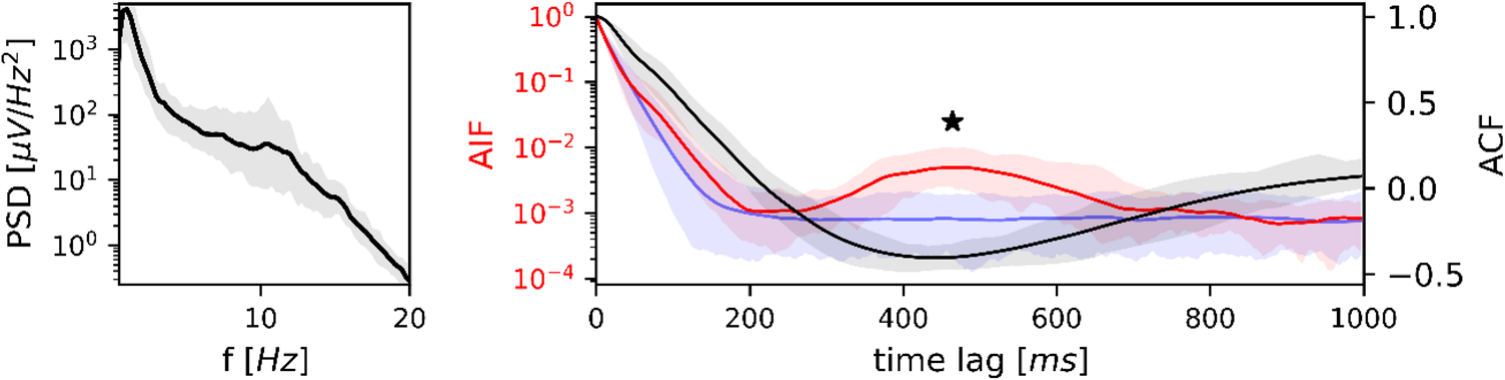
shows the frequency analysis for sleep stage N3. The EEG spectrum in N3 was dominated by delta activity (0.5-3 Hz) which accounted for the majority of total data variance (84% of the PSD area). The first ACF local maximum was located outside the analysed area (> 1000 ms), the first ACF local minimum was at 440 ms (black curve), close to the first AIF peak at 464 ms (red curve). The AIF peak at 464 ms clearly exceeded the Markov confidence interval (blue area), indicating a large amount of shared information at 1.1. Hz, compared to surrogate Markov data. The AIF peak test confirmed the statistical significance of the peak (*p < 0.001*), relative to Markov surrogate AIF (blue curve)

Figure 6 PSD, ACF and AIF of microstate sequences in sleep stage N3. Left: average power spectral density (*n* = 19) in semi-logarithmic coordinates. Right: average EEG autocorrelation function (black) and microstate autoinformation function (red). Markov surrogate data are shown as the 95% confidence interval (blue shaded area) and mean surrogate AIF (blue line). The PSD is dominated by high delta power and a smaller peak at 10.3 Hz. The ACF has its first local minimum at 440 ms (1.1 Hz). The AIF peak lies at 468 ms (1.1 Hz) and is significant with respect to the Markov confidence interval while the surrogate AIF has a monotone decay; the AIF peak test was significant for the AIF peak (marked by asterisk). PSD, ACF and AIF are shown along with their confidence intervals (5–95%).

## Discussion

### Summary of Findings

To better understand microstate dynamics in different vigilance states, we analyzed temporal properties of microstate sequences during wakefulness and NREM sleep across several time scales. Ordered by time scales, our findings are summarized as follows:

- For deeper sleep stages, microstate dynamics slow down, as demonstrated by increasing mean durations for all microstate classes. Slowing occurs continuously across the sleep stages as shown by the increasing relaxation time of the transition matrix.
- Explicitly testing Markov properties on very short time scales reveals that microstate sequences in wake, N1 and N2 have memory effects extending for at least two sampling intervals (8 ms). In N3, however, n=10/19 microstate dynamics simplified and could be described by a second-order Markov model.
- Microstate sequences became more predictable in deeper sleep stages, quantified by a decreasing entropy rate over a 24 ms time window. This property was highly correlated with the transition matrix relaxation time.
- Frequency analysis of microstate sequences for time lags up to 1000 ms showed that microstate sequences can follow EEG alpha, theta, delta and sleep spindle frequencies in different vigilance states, a property not predicted by the transition matrix approach.

### Microstate Topographies in Wakefulness and Sleep

Using the well-studied modified k-means clustering algorithm (Pasqual–Marqui et al. 1995), we found microstate topographies similar to those described in most wakeful rest studies (Koenig et al. 2002; Michel and Koenig 2018). The maps presented here are based on the same data set analyzed in Brodbeck et al. (2012), but using a different preprocessing strategy. Instead of 1–40 Hz band-pass filtering and the TAAHC clustering algorithm (Brodbeck et al. 2012), we chose a frequency range of 0.5–20 Hz and the modified k-means algorithm. The aim was to include broader delta frequency band content which may contain relevant information in deeper sleep stages (N3), and to exclude higher frequencies possibly contaminated by noise. The upper frequency band limit of 20 Hz is a common choice in resting state microstate studies (Koenig et al. 2002; Lehmann et al. 2005; Pasqual-Marqui et al. 2014; Diaz et al. 2016).

We chose the group maps in agreement with the canonical maps defined in the literature (Michel and Koenig 2018), even though individual trials of the permutation algorithm sometimes gave non-canonical microstate maps, for instance maps with a fronto-occipital symmetry axis. Compared to the canonical maps, their GEV values differed by less than 0.5%, only. Though label-dependent quantities such as MMD and GEV per map are dependent on the choice of the group maps, the label-independent values (T_relax_, entropy rate, and the autoinformation function) should not be affected, as suggested by our previous work (von Wegner et al. 2018).

We reproduced the finding that microstate map D has a more circular pattern in sleep compared to wakefulness, a feature discussed in detail in Brodbeck et al. (2012). Brodbeck et al. (2012) reported that their N2 group maps had the lowest correlation with the wake maps, an effect mainly driven by the more circular pattern of map D. We could reproduce this finding under the current preprocessing strategy, and made the additional observation that map D was not only different in N2 (correlation coefficient to W: 53.8%) but also in N1 (62.2%). There is evidence that even subtle alterations in map geometries indicate significantly different dynamic functional connectivity patterns (Abreu et al. 2021). Thus, the topography of microstate class D appears to be the best indicator of light sleep, and the actual network underlying this topography is likely to be different from the network corresponding to class D in wakefulness. Thus, the comparison of microstate class D statistics between conditions which may also reflect different vigilance levels should be interpreted accordingly, they may not correspond to the same functional network.

In contrast to Brodbeck et al. (2012), we did not reproduce the finding that map B was the most prominent in terms of GEV in sleep stage N2, and that map C predominated in all other vigilance states. In our analysis, map C had the largest GEV values in all vigilance states including N2 (W: 18.2%; N1: 20.9%; N2: 26.7%; N3: 26.5%). Similar observations were recently reported for microstate distributions in slow-wave sleep (Xu et al. 2020) and in a sleep study using a five microstate decomposition (Bréchet et al. 2020).

The details of our data processing pipeline may have contributed to the differences described as the band-pass filter was different from the one used in Brodbeck et al. (2012), which affects the smoothness of the obtained surface topographies, and thus influences the goodness-of-fit between data vectors and microstate maps as well as the final microstate statistics.

In Brodbeck et al. (2012), we used the modified cross-validation criterion (Pasqual-Marqui et al. 1995) to compute the optimum cluster number of K = 4. The pre-processed data used in this manuscript gave an optimum cluster number of K = 4 for W and N1, and K = 5 for N2 and N3. The supplementary file to this article shows the results for K = 5 for all vigilance states. The important observation is that the key findings of this article, as shown in Figs. 2, 3, 4, 5 and 6, are also observed for K = 5. This confirms our earlier findings that the microstate class-independent and entropy-based metrics used here are stable against variations in the number and geometry of microstate maps (von Wegner et al. 2018). An interesting observation is that K = 5 clusters revealed the additional microstate map E, following the labeling in Custo el al. (2017), for W and N1, and the map F for N2 and N3. This relates to recent research into propofol-induced vigilance loss (Shi et al. 2020; Artoni et al. 2022), where K = 5 microstate maps were found to be optimal as well. These authors, however, found map F in their wakeful baseline conditions. This observation can partially be explained by the fact that some subjects in the publicly available data set used by Shi et al. (2020) show clear signs of drowsiness. An additional map was also found in narcolepsy patients (Kuhn et al. 2015). It remains an open question whether the need for more than four microstate maps is a generalizable feature of reduced vigilance and consciousness. An unresolved problem is that the assignment to microstate classes becomes more difficult when more classes are used. Our supplementary Fig. S1 illustrates this for sleep stage N3, where the assignment of microstate classes C, D is ambiguous, and the last map in W and N1 appears to be class E according Custo el al. (2017), but the map E differences between W and N1 are not negligible. Our proposed solution to this problem is to rely more on microstate metrics that are robust against changes in the number and labeling of microstate maps. We believe that the methods used in this manuscript might be helpful in achieving this goal.

Overall, our results confirm that microstate topographies A–C are relatively robust features of spontaneous brain activity in wake and NREM sleep, whereas map D and additional maps in the case of larger cluster numbers (suppl. Fig. S1) show considerable variability. On the negative side, individual map statistics can be affected by preprocessing and the role of individual maps is still unclear. Moreover, the same map label (e.g. class D) may represent different network configurations when compared between vigilance states. The appearance of a microstate map with a high similarity to one of the known geometries A–G (Custo el al. 2017) should not, in general, be interpreted as the same functional brain network if the experimental conditions are different. Despite their similarity, we refrain from interpreting our maps A–C in N2 and N3 sleep stages as correlates of the same active cognitive processes that were studied in (Seitzman et al. 2017) for instance, as the behavioural states (engaging in a cognitive task vs. sleep) suggest fundamental differences in brain state and function. Given the limitations of a map-centered interpretation, we directed our focus on temporal correlations in the following.

### Slowing of Microstate Dynamics and loss of Complexity

Sleep-related EEG changes are diverse, a common feature being an increase in the proportion of lower frequencies, although individual higher frequency events such as sleep spindles also occur. We observed EEG slowing for deeper sleep stages in the GFP time course, i.e. in the frequency of GFP peaks (local maxima) per second. Compared to the previous study by Brodbeck et al. (2012) (W: 31.2; N1: 31.8; N2: 27.6; N3: 13.4/s), our PPS values were lower (W: 18.5; N1: 16.6; N2: 15.4; N3: 11.5/s), due to the lower high-frequency cut-off of the band-pass filter in our study (20 Hz vs. 40 Hz).

To correctly interpret the mean microstate durations with respect to the literature, we must emphasize the fundamental difference between our algorithm and many other microstate studies. We fit the best matching microstate at each EEG sampling time point (von Wegner and Laufs 2018; von Wegner et al. 2021), whereas many other researchers calculate this fit at GFP peaks only, and interpolate the labels between GFP peaks (e.g. Lehmann et al. 1987, 1998; Koenig et al. 2002; Brodbeck et al. 2012). The latter approach has a smoothing effect, as switches between microstate labels are only allowed to happen in the GFP troughs between the peaks, imposing a lower limit on microstate durations, and an upper limit on the observed microstate frequencies. For example, a PPS value of 20/s implies that the interpolation method renders microstate durations below 50 ms very unlikely, by construction. Our method uses minimal assumptions and therefore allows us to observe the full bandwidth of temporal dynamics. Recent studies by us and others could show that the dynamics between GFP peaks contain relevant information about the actual continuous dynamics of the cortical electric field (Mishra et al. 2020; von Wegner et al. 2021).

A consequence of our approach is that the mean microstate durations are significantly shorter than those reported in many classical microstate papers. For all vigilance states, the MMD values in the present study were shorter than those found in Brodbeck et al. (2012), where the interpolation method was used: 21.9 vs. 44.8 (W), 28.3 vs. 44.9 ms (N1), 31.4 vs. 57.9 ms (N2), and 53.3 vs. 81.4 ms (N3). Between sleep stages, we found a higher MMD in N1 compared to W, while Brodbeck et al. (2012) found almost no difference. The MMD difference of microstates A, C and D between N1 and N2 was not significant in our study. Both studies agree that the largest MMD increase exists between W and sleep stage N3, with a MMD in N3 between two times (Brodbeck et al. 2012) and two-and-a-half times (+ 143% in our study) higher than in wakefulness.

Our additional metrics, however, demonstrate that the transition from W to N3 is accompanied by a smooth, continuous slowing of microstate dynamics. The relaxation time of the transition probability matrix, a time constant that describes how fast a stochastic process returns to equilibrium after a perturbation, captures the continuous aspects of microstate dynamics. Its inverse, the spectral gap is shown in Fig. 2 and shows a monotone decay across the vigilance states. The limitation of the relaxation time approach is its temporal scope, which it inherits from the transition matrix, i.e. it only considers single time step dynamics (*t → t + 1*).

We therefore included further time steps in our analysis using the (finite) entropy rate with a time window of 24 ms. The (finite) entropy rate behaved in exactly the same way as the relaxation time, as illustrated in Fig. 2. With deepening sleep stage, the entropy rate decreased continuously, and clearly correlated with the microstate duration. The entropy rate of a microstate sequence measures the amount of surprise about the next microstate, given knowledge about its history (conditional entropy). The decreasing entropy rate during sleep means that more information about the next brain state is contained in the immediate past brain activity, rendering network transitions more predictable, and less complex. The complexity of the microstate process however can be defined in different ways and cannot be interpreted without taking into account the underlying frequency at which these processes run. When oscillatory brain activity with a given complexity slows down by a given factor, its entropy rate will decrease accordingly, yielding a more predictable process. However, the process would still be able to encode the same amount of information per oscillation cycle, it would just be stored over a longer time window. Therefore, the notion of complexity has to be used carefully, and in a well-defined context. A different definition of microstate sequence complexity (Lempel–Ziv compression), correcting for the underlying “carrier frequency” was used by Tait et al. (2020) and a similar approach was used by Artoni et al. (2022). Direct comparison with our results is difficult as these studies remove all duplicate microstate labels before compression. This leads to a highly non-uniform distortion of the time axis, as the sequences ‘AAAABAB’ and ‘ABABBBB’, for example, would be identical after removal of duplicate labels. They are both transformed into ‘ABAB’ before complexity is computed. Our approach however, focuses on time series features for which the integrity of the time axis is essential. Despite the methodological differences it is interesting to observe analogies such as the decrease of Lempel–Ziv complexity during deeper states of propofol-induced anesthesia (Artoni et al. 2022), and the reduced entropy rate during deep sleep found in this study. Direct comparison of different methods on the same data set would give further insights.

Another assessment of microstate sequence structure over short time scales is to test their deviation from low-order Markov models, as tested by formal Markovianity statistics. Earlier analyses of wakefulness EEG demonstrated that microstate sequences show very strong deviations from Markovianity (von Wegner et al. 2017). Similar observations were obtained here for W, N1, and N2, where the Markov property was rejected in all data sets. We did not expect to find that n = 10/19 N3 recordings gave test results compatible with a second-order Markov process, as compared to a third-order process. In other words, 10 out of 19 microstate sequences had *t → t + 1* transition probabilities that were fully predicted by the microstates at time points *t* and *t-1*, whereas addition of the microstate label at *t-2* had no further predictive effect.

This observation, together with the lower microstate entropy rate, speaks for a more restricted, and therefore more predictable trajectory of the underlying neuronal ensembles in deeper sleep stages. We use the term trajectory in analogy to dynamical systems models of neuronal activity. A single microstate map summarizes the simultaneous activity of a very large number of neurons. From a modelling perspective, the temporal evolution of this large neuronal ensemble is a multivariate dynamical system (Deco et al. 2008). In this picture, microstate sequences can be seen as a discrete approximation to the continuous dynamics described by a vector of 30 (number of EEG channels) coupled voltage variables. In our case, the microstate algorithm partitions the state space into four discrete regions, each region being represented by a microstate map. The lower entropy rate of microstate sequences in sleep corresponds to a more predictable trajectory in the discretized space, and it can be hypothesized that the non-discretized, continuous trajectory would also be more predictable. During complex cognitive tasks, the largest microstate entropy rates have been observed in subtasks with the highest cognitive workload and the lowest degree of cognitive control (Jia et al. 2021). The current results extend these observations while pointing in the same direction, as they suggest a further decrease in cognitive workload with deepening sleep. Our results also suggest that other than cognitive control mechanisms can restrict the degrees of freedom with which microstates are generated. We hypothesize that sleep-specific network mechanisms, including subcortical and brainstem activity, might restrict microstate dynamics in sleep, as opposed to cortical cognitive control mechanisms during cognitive task execution. It can be hypothesized that a partial isolation from external sensory stimuli as well as the synchronized slow wave activity organize and restrict cortical activity and thus, microstate dynamics.

### Periodicities

Finally, the hallmark of EEG recordings are oscillations in different frequency bands, and the frequency composition of ongoing EEG in wakefulness and sleep shows marked differences (Rechtschaffen and Kales 1968; American Academy of Sleep Medicine 2007). The relationship between microstates and EEG frequencies is still unclear and has been addressed in several studies. Britz et al. (2010) reported a lack of correlation between the power of EEG frequency bands and microstate prevalence during wakefulness. Comsa et al. (2019) detected an association of microstate D with EEG functional connectivity in the theta band during the transition from wakefulness to drowsiness. Shi et al. (2020) investigated the instantaneous EEG frequency during the lifetime of individual microstates and found that five microstate classes had very similar marginal EEG spectra during propofol sedation. Abreu et al. (2021) used topographic time-frequency decomposition, a technique which yields a time-frequency plot for each microstate map (Koenig et al. 2001), and reported characteristic EEG frequency spectra for ten different microstate maps. None of these techniques, however, tests for the periodic appearance of the microstate labels themselves. We introduced and validated a technique for microstate frequency analysis in a resting-state EEG study set (von Wegner et al. 2017), and found that alpha oscillations (10 Hz) were linked to periodic microstates with twice that frequency, i.e. a minimum recurrence interval of 50 ms. Frequency doubling was explained by microstate maps matching the EEG topography twice per alpha cycle, due to the fact that alpha oscillations invert their polarity every half-cycle (50 ms), and the polarity-ignoring property of the microstate fitting algorithm (Lehmann 1971).

The preprocessing applied to microstate sequences is expected to affect their periodic features. Several smoothing strategies have been suggested for EEG microstate sequences and they are used with different parameter settings by different authors. Two prominent strategies are (i) regularized smoothing (Pascual-Marqui et al. 1995), and (ii) ‘interpolation’ of microstate labels between GFP peaks, i.e. using the microstate class fitted to the closest GFP peak (e.g. Lehmann et al. 1987, 1998; Koenig et al. 2002; Brodbeck et al. 2012). We expect that any smoothing strategy will attenuate or even abolish periodic sequence features when the smoothing time window approaches the corresponding oscillation period. In wakefulness, a smoothing window size close to 50 ms would most likely affect periodicities. Smoothing time scales close to or even beyond the periodicity time lags observed here have been used in the past (Tomescu et al. 2014).

#### Wakeful Rest

In wakefulness, we reproduced clearly defined oscillatory microstate dynamics related to the underlying alpha frequency band as described in von Wegner et al. (2017). The mutual information (autoinformation) of microstate sequences had peaks coinciding with all local extrema of the autocorrelation function of the underlying EEG. As entropy values are non-negative, local maxima of the AIF occur at the locations of local minima and maxima of the ACF. This observation confirms that the functional networks captured by microstates activate periodically during wakefulness.

#### Sleep Stage N1

Microstate frequency analysis shows that the differences between W and N1 go beyond the change in microstate D topography and general EEG slowing. Even though brain activity in N1 can effectively be described by the four microstate classes, the regular periodic activation profile is almost completely lost in a brain state that corresponds to drowsiness. This observation lifts the EEG definition of sleep stage N1 to the network level. Sleep stage N1 is assigned when less than 50% of a 30 s EEG segment shows the posterior dominant rhythm, usually in the alpha band, which is replaced by low amplitude mixed frequency activity (American Academy of Sleep Medicine 2007). Our analysis shows that the loss of the occipital alpha rhythm is accompanied by a temporal disordering (loss of periodicity) on the level of EEG microstates. It can therefore be hypothesized that loss of microstate periodicity may be a useful marker in other types of drowsiness, as caused by pharmacological agents or neurological conditions. The observation that microstate sequences can be linked to N1 theta oscillations, although observed in only one subject, is a novel finding, demonstrating that sleep-related networks can also activate periodically in the theta frequency range.

#### Sleep Stage N2

Microstate properties averaged over sleep stage N2 segments showed further slowing of microstate dynamics, but no spectral peaks. Isolated sleep spindle segments, however, showed periodic microstates linked to the sleep spindle frequency of 12.5 Hz. Similar to other frequency bands, the principal AIF latency coincided with half the spindle oscillation length (40 ms, or 25 Hz), suggesting that the mechanisms leading to frequency doubling, as detailed above, are also valid for sleep spindle oscillations. Sleep spindle statistics were different from other frequency bands in that the microstate AIF peaks did not exceed the Markov confidence interval, indicating that the absolute information content contained in these oscillations was not larger than that contained in the equivalent Markov model. In line with this observation, the explicit Markov tests of these short microstate sequences showed no deviation from a Markov process. However, this effect is mainly due to the low number of samples used. We tested this by using short higher-order Markov surrogates. These higher-order properties could not be detected by the Markovianity tests in sequences as short as sleep spindles, whereas longer sequences were classified correctly (analysis shown in supplementary Table S3). These statistical effects might also have played a role in early microstate studies where 15 s microstate sequences were classified as Markovian (Wackermann et al. 1993).

The statistical significance of sleep spindle associated microstate oscillations is proven by the AIF peak test that quantifies the observation that none of the Markovian surrogates had AIF peaks. The finding of periodic microstates during sleep spindles provides an interesting link between sleep spindle-linked EEG spiral waves (Muller et al. 2016) and the rotating EEG phase patterns we identified as a basic mechanism underlying periodic microstates during wake EEG (von Wegner et al. 2021).

#### Sleep Stage N3

Network activity during sleep stage N3, which is characterized by delta frequencies (0.5-3 Hz), shows the same qualitative behavior as found in the other vigilance states, i.e. periodic microstates that are closely linked to the EEG frequency spectrum, but transposed into the delta frequency band. A recent study identified the four canonical microstates in slow-wave sleep and mapped them to well-known functional networks (auditory, executive control, saliency) defined by independent component analysis from fMRI data (Xu et al. 2020). Combining these results with our study, we can predict that fMRI-defined networks should also activate periodically, with an oscillation length of approximately 1 Hz. The time scale of these oscillations is still faster than the sampling rate of most fMRI sequences, which makes experimental verification challenging. Since fMRI resolves at timescales that depend on the repetition time (TR) of the scanning as well as on the blood flow rate (Demetriou et al. 2018), we believe that microstate dynamics add relevant information about the neurobiology of sleep by revealing network behavior in the sub-second range.

An earlier analysis of the data set presented here revealed that increasing delta activity was correlated with a loss of functional connectivity within occipital areas, as well as between occipital and central areas (Tagliazucchi et al. 2013) while other studies found disintegration of mediofrontal functional networks during sleep (Horovitz et al. 2009; Bréchet et al. 2020). Since different brain areas have to activate in a phase-locked manner to produce a stable microstate topography (Koenig and Valdés-Sosa 2018), these connectivity changes might explain the altered microstate map D topography in sleep, where occipital and frontal areas appear to be dissociated from the microstate D network. The exact mechanisms underlying these connectivity changes remain to be clarified. Early cellular studies suggested a role of the thalamus in generating and synchronizing cortical delta oscillations but also pointed out that the cortex can generate these frequencies without thalamic input (Amzica and Steriade 1998). On the other hand, we know that the thalamus disengages from supratentorial networks in N3, instead joining a functional module which contains the cerebellum (Tagliazucchi et al. 2013). This functional uncoupling of the thalamus from the cortex would rather suggest less synchronized cortical networks in N3, contradicting our empirical evidence from microstate analysis. This line of arguments shows that the insights obtained from different modalities (cellular recordings, surface EEG and fMRI) are not easily integrated in a framework using simple models of regional (de-)activation and functional coupling. However, within the EEG microstate framework, our analysis demonstrates that 1 Hz oscillations are a robust and statistically significant phenomenon.

### Conclusion

EEG microstates are dynamic and periodic phenomena and occur at variable frequencies that are closely related to the dominant EEG frequencies in different vigilance states. We demonstrated that microstate dynamics continuously slow down across the NREM sleep stages and that oscillatory microstate dynamics linked to alpha, theta, delta and sleep spindle frequencies are observed whenever EEG signals at the sensor level show defined spectral peaks. These findings suggest that the oscillations observed at individual electrodes extend to oscillatory activity of large-scale brain functional networks.

## Supplementary Information

Below is the link to the electronic supplementary material.

Supplementary material 1 (DOCX 594.3 kb)

## Data Availability

The original EEG datasets analyzed in this study are not publicly available due to conflicts with the underlying Ethics approval. Processed data and analysis scripts are available upon request.
