# Supplementary material for: Frequency Analysis of EEG Microstate Sequences in Wakefulness and NREM Sleep
Source: Brain Topogr. 2023 May 30;37(2):312–28. doi: 10.1007/s10548-023-00971-y (PMC11374823; doi:10.1007/s10548-023-00971-y)
Supplement: Supplementary file 1 — Supplementary material 1 (DOCX 594.3 kb) [file 10548_2023_971_MOESM1_ESM.docx]

**SUPPLEMENTARY DATA**

**1. ANOVA statistics**

Table S1 shows the details of the ANOVA tests for the quantities GFP peaks per second (PPS), mean microstate duration (MMD) and global explained variance (GEV).

| Table S1  One-way ANOVA results: Statistical differences between vigilance states (PPS, MMD) and between microstate maps (GEV). | | | | |
| --- | --- | --- | --- | --- |
|  | dfc | dfe | F-ratio | p value |
| PPS | 3 | 111 | 197.4 | < 0.001 |
| MMD | 3 | 111 | 291.0 | < 0.001 |
| MMD_A_ | 3 | 111 | 187.9 | < 0.001 |
| MMD_B_ | 3 | 111 | 161.2 | < 0.001 |
| MMD_C_ | 3 | 111 | 154.7 | < 0.001 |
| MMD_D_ | 3 | 111 | 205.5 | < 0.001 |
| GEV_W_ | 3 | 124 | 7.67 | < 0.001 |
| GEV_N1_ | 3 | 124 | 11.42 | < 0.001 |
| GEV_N2_ | 3 | 124 | 23.43 | < 0.001 |
| GEV_N3_ | 3 | 72 | 15.60 | < 0.001 |
| GEV_SLS_ | 3 | 124 | 4.58 | 0.004 |
| All ANOVAs were performed on a significance level of *α = 0.05*. | | | | |

**2. Microstate analysis for K=5 clusters, group and grand mean maps**

In the main text, we report the results for *K* = 4 microstates, as used for the same dataset in (Brodbeck et al. 2012). When the modified k-means algorithm is run with *K* = 3...8 clusters for each sleep stage, and each clustering result is assessed with the modified cross-validation criterion (Pasqual-Marqui et al. 1995), the optimum number of clusters for W and N1 is *K* = 4, and *K* = 5 for N2 and N3. Moreover, there is a choice between the use of microstate maps for each condition, i.e. for each sleep stage, or using grand mean microstate maps obtained across all sleep stages. A recent preprint presented evidence for an increased chance of false positive results when some microstate statistics are compared based on condition-wise clustering (Murphy et al. 2022). To test the robustness of our main results against these variations, we here show the clustering results. Fig. S1 shows the microstate maps for K=5 clusters in each sleep stage (W, N1, N2, N3), and the grand mean maps in the last row (GM). Using the microstate assignment defined in Custo et al. (2017), the sleep stage-specific microstate classes for K=5 correspond to A, B, C, D, and E in stages W and N1, and to microstate classes A, B, C, D, F in N2, N3, and the grand mean (GM) clustering. This corresponds to the maps in Fig. S1 from left to right.


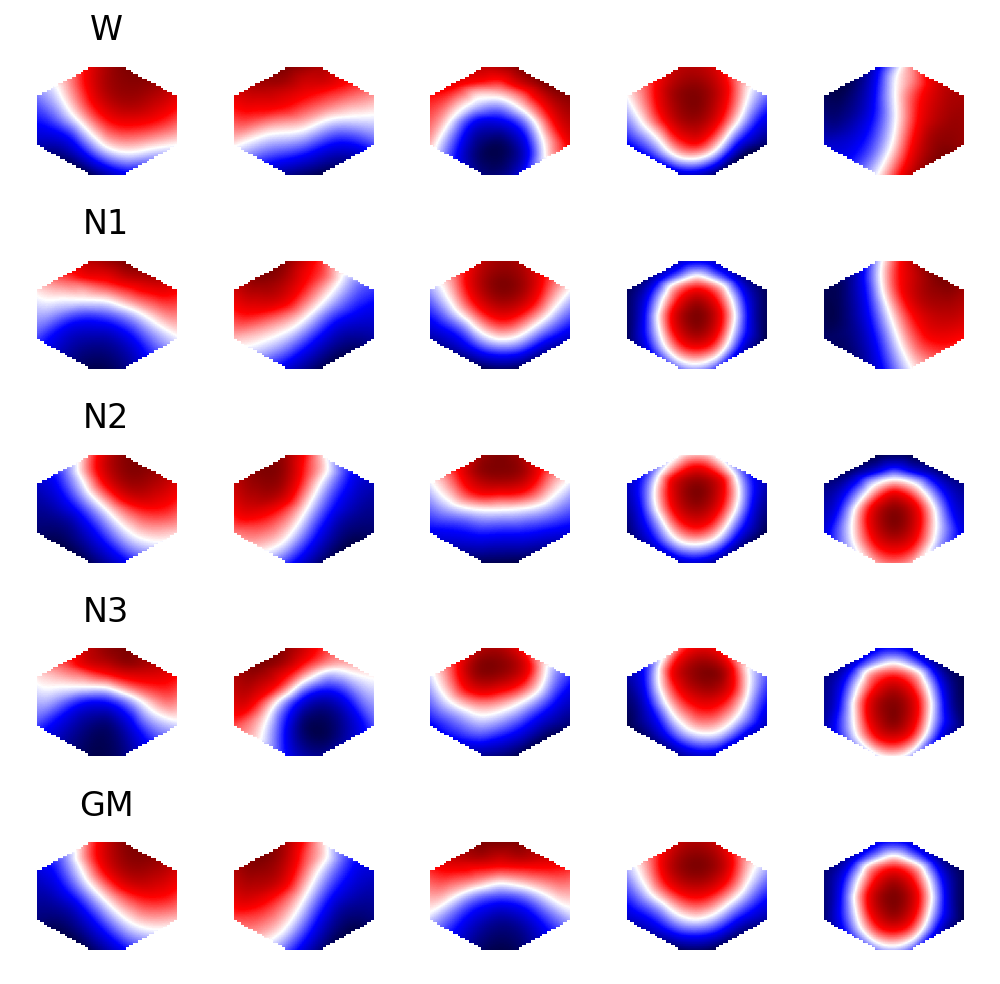


**Fig. S1** Microstate maps for *K* = 5 clusters from the modified k-means algorithm.

The conclusions derived from Fig. 2 in the main manuscript are reproduced for *K* = 5 microstate maps in Fig. S2. Results for sleep stage-specific maps are on the left and for grand mean maps on the right. The monotone relationship between mean microstate durations and a) the entropy rate, and b) the spectral gap, is also found for *K* = 5 and independent of the underlying maps. Similar to *K* = 4, sleep stage N3 forms a separate cluster while W, N1 and N2 show more overlap.


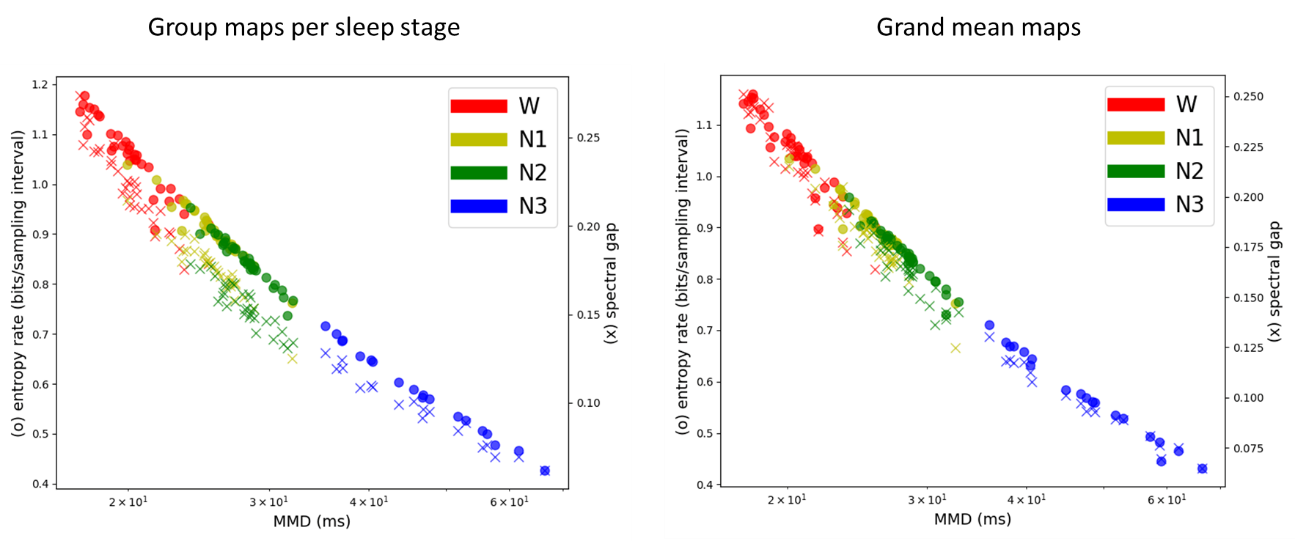


**Fig. S2**: Mean microstate durations vs. entropy rate and spectral gap. Microstate sequences were computed from sleep-stage specific maps (left) and with grand mean maps (right). The features observed in Fig. 2 in the main text are reproduced with good agreement for *K* = 5 clusters.

Likewise, periodic features of the autoinformation functions (AIF) in W, N1, N2, and N3 do not depend on the choice *K* = 4 vs. *K* = 5, or on the level of clustering (sleep stage vs. grand mean). The results for *K* = 5 are summarized in Fig. S3.


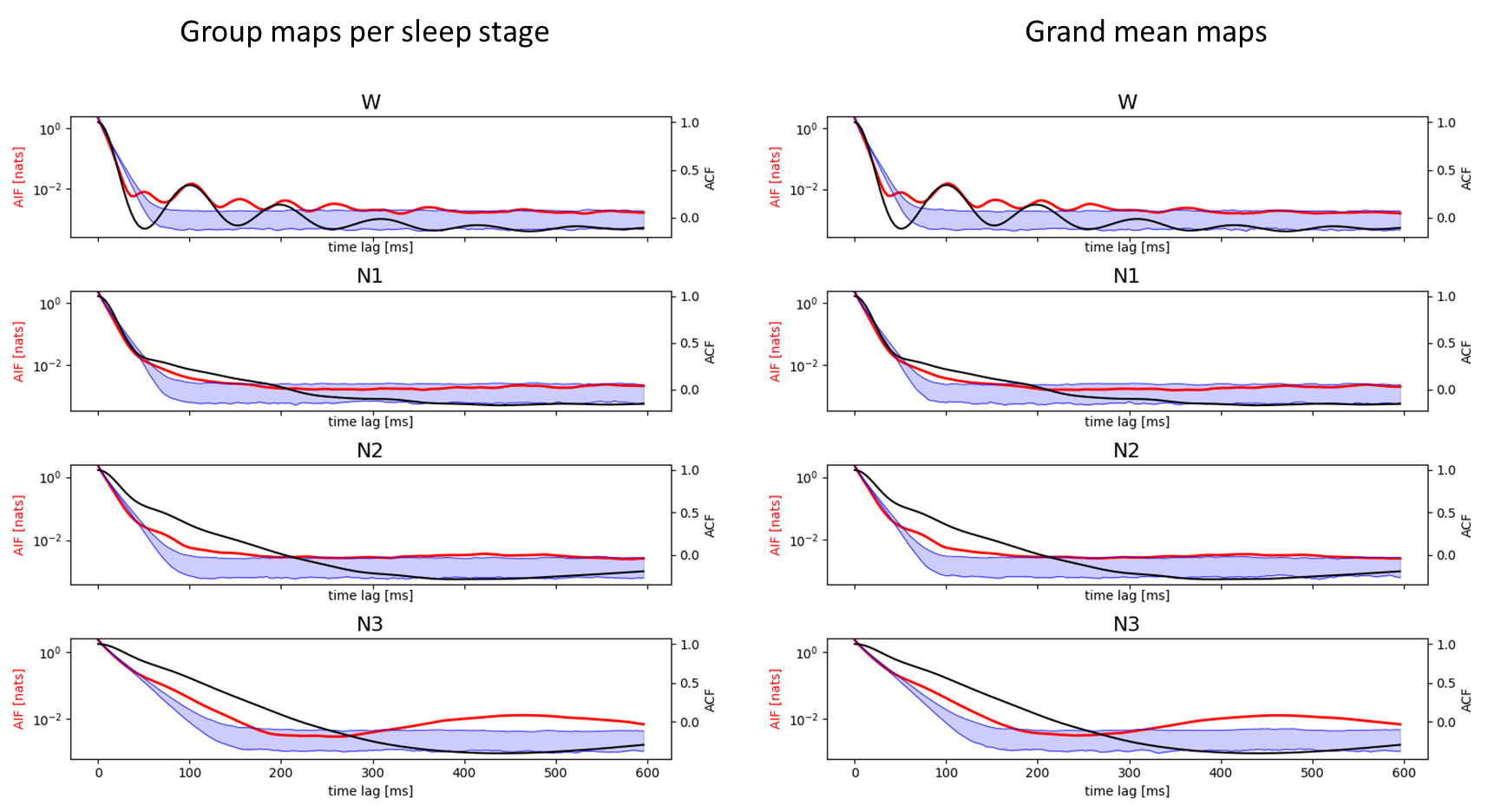


**Fig. S3** The AIFs obtained for *K* = 5 microstates across the vigilance states W, N1, N2, N3 follow the same pattern as those obtained for *K* = 4. Left: microstate sequences from sleep-stage specific microstate maps. Right: microstate sequences from grand mean maps.

**3. Markov tests for *K* = 5**

The Markov test results for *K* = 5 microstates and sleep stage-specific microstate maps are concordant with *K* = 4, with even stronger evidence for low-order Markovianity in sleep. The chance of accepting the null hypothesis of a second-order Markov process was increasingly likely when moving from W to N3. The results are shown in Table S2.

| Table S2  Markov tests for microstate sequences with *K* = 5 microstate classes, and separate microstate maps for each sleep stage. Rows: sleep stages W, N1, N2, N3. Columns: Markov-*k* refers to chi-square tests for *k*-th order Markovianity. | | | |
| --- | --- | --- | --- |
|  | Markov-0 | Markov-1 | Markov-2 |
| W | 32/32 | 32/32 | 32/32 |
| N1 | 32/32 | 32/32 | 23/32 |
| N2 | 32/32 | 32/32 | 10/32 |
| N3 | 19/19 | 19/19 | 1/19 |
| Data: the number of rejected null hypotheses after Bonferroni correction (*α = 0.05*). | | | |
|  | | | |
|  | | | |

Table S3 contains the results for the corresponding Markov tests on microstate sequences computed with K=5 grand mean microstate maps. The results are compatible with the results of sleep stage-specific clustering for both K=4 (see main text), and K=5 (Table S2).

| Table S3  Markov tests for microstate sequences with *K* = 5 microstate classes (grand mean maps). Rows: sleep stages W, N1, N2, N3. Columns: Markov-*k* refers to chi-square tests for *k*-th order Markovianity. | | | |
| --- | --- | --- | --- |
|  | Markov-0 | Markov-1 | Markov-2 |
| W | 32/32 | 32/32 | 32/32 |
| N1 | 32/32 | 32/32 | 24/32 |
| N2 | 32/32 | 32/32 | 11/32 |
| N3 | 19/19 | 19/19 | 1/19 |
| Data: the number of rejected null hypotheses after Bonferroni correction (*α = 0.05*). | | | |
|  | | | |
|  | | | |

**4. Markov test power for short N2 microstate sequences**

Testing the Markov property of microstate sequences representing sleep spindles in sleep stage N2 resulted in the rejection of 32/32 sequences for zero-order Markovianity, 1/32 sequences for first-order Markovianity, and 0/32 sequences for second-order Markovianity (main text). As the power of statistical tests decreases with sample size and sleep spindles are relatively short, we estimated the power of the applied Markov tests with surrogate sequences of known Markov order. For each of the *n* = 32 microstate sequences from sleep stage N2, we calculated 10 surrogate sequences with known Markov order *k* = 3. The corresponding third-order transition matrix $P(X_{t}|X_{t-1},X_{t-2},X_{t-3})$ was derived from the underlying N2 microstate sequence. Next, to compare the Markov test power of long and short sequences, we extracted 10 sub-sequences from each surrogate, each of length 2 seconds.

The results are shown in Table S4. For the full length third-order Markov surrogate sequences (same length as the N2 sequences), all Markov tests of order 0, 1, and 2 were rejected (true positives). For the short sequences (2 s), however, only the zero-order Markov test was free from false negatives. First- and second-order Markov test results were all false negatives. The tests should have rejected the null hypotheses as the surrogates were of order k=3 by construction, but the respective null hypotheses were accepted. This shows that the test results suggesting a Markov order k=2 for sleep spindles are not reliable. The true Markov order of sleep spindle microstate sequences, if it exists, remains unknown.

| Table S4  Reliability of Markovianity tests for short segments: For each N2 microstate sequence (*n* = 32), ten Markov surrogate sequences of order 3 were created. From each of those, ten random segments of length 2 s were extracted. The higher order Markov surrogates (*n* = 320) and random short segments (*n* = 3200) were tested for the Markov properties of order 0, 1 and 2. The table shows the number of null hypothesis rejections in both groups. | | |
| --- | --- | --- |
|  | Markov surrogates | Random short segments (2 s) |
| Markov-0 | 320/320 | 3200/3200 |
| Markov-1 | 320/320 | 0/3200 |
| Markov-2 | 320/320 | 0/3200 |
| Statistical significance was tested for *α = 0.05*. All p values were Bonferroni corrected. | | |

**References**

Custo A, Van De Ville D, Wells WM, Tomescu MI, Brunet D, Michel CM (2017) Electroencephalographic Resting-State Networks: Source Localization of Microstates. Brain Connect 7(10):671-682.

Pasqual-Marqui RD, Michel CM, Lehmann D (1995) Segmentation of brain electrical activity into microstates: model estimation and validation. IEEE Transactions on Biomedical Engineering 42(7):658-665.

Murphy M, Wang J, Jiang C, Wang L, Kozhemiako N, Wang Y, the GRINS consortium, Pan JQ, Purcell SM (2022) Bias in group-level EEG microstate analysis. bioRxiv preprint <https://www.biorxiv.org/content/biorxiv/early/2022/12/14/2022.11.07.515464.full.pdf>
